# Early-stage intercropping modulates rhizosphere soil properties, microbiome composition, and photosynthetic physiology of *Phellodendron chinense*: a comparative analysis of five cultivation systems

**DOI:** 10.3389/fpls.2026.1882609

**Published:** 2026-09-04

**Authors:** Chuan Xie, Jiaojiao Wu, Peng Song, Xiuchan Xiao, Xinyuan An, Huawei Wu, Zhipeng Sun

**Affiliations:** 1 Ecological Conservation, Restoration and Resource Utilization in Forest and Wetland Key Laboratory of Sichuan Province, Sichuan Academy of Forestry, Chengdu, China; 2 School of Materials and Environmental Engineering, Chengdu Technological University, Chengdu, China

**Keywords:** intercropping, *Phellodendron chinense*, photosynthetic physiology, plant-soil feedback, rhizosphere microbiome

## Abstract

**Introduction:**

*Phellodendron chinense* is an important medicinal plant in China. However, continuous monoculture has caused soil degradation and physiological constraints, which have become a key bottleneck limiting its sustainable production. Intercropping with companion plants is an effective approach to break this dilemma.

**Methods:**

We selected five representative cultivation systems commonly practiced of *Phellodendron chinense* and systematically measured their rhizosphere soil properties, microbial community composition, and leaf gas-exchange traits. The overarching goal was twofold: (i) to characterize the intercropping-induced variations in these three dimensions (soil properties, microbial communities, and photosynthetic physiology, and (ii) to elucidate the coupling relationships among these three dimensions.

**Results and Discussion:**

The results demonstrated that total nitrogen(N), total sodium(Na), and magnesium(Mg) contents in the T4 and T2 patterns were significantly higher than those in other patterns. The T2 and T4 patterns significantly enhanced microbial community α-diversity. In contrast, the T3 pattern caused significant soil acidification (pH 4.69) and elevated the relative abundance of the tolerant phylum Chloroflexi. T4 and T2 patterns significantly alleviated stomatal limitations in *P. chinense*, achieving optimal net photosynthetic rate(Pn) and stomatal conductance(Gs). Correlation analysis showed that Pn was significantly and positively correlated with soil P and Mg contents, while Gs was significantly and positively correlated with soil Na content, and N and Na were the critical factors affecting the structural variation of bacterial and fungal communities. Overall, this study demonstrates that intercropping promote soil nutrient accumulation enhances rhizosphere microbial diversity to facilitate the alleviation of photosynthetic physiological limitations. Intercropping *P. chinense* with bamboo or tea synergistically mitigates the host’s photosynthetic physiological limitations through a belowground positive feedback pathway. These findings provide an important theoretical basis for the efficient and sustainable cultivation of this rare medicinal plant.

## 1 Introduction

*Phellodendron chinense* Schneid is a deciduous, dioecious tree of the Rutaceae family, reaching up to 15 m in height, with a thick corky bark and a bitter yellow inner bark. It thrives in warm, humid climates and deep, well-drained and is naturally distributed in montane mixed forests across central and southern China (800–1500 m a.s.l.) (Luo et al., 2025; Shi et al., 2023). *P. chinense* is a traditional bulk medicinal herb in China; its bark is rich in bioactive compounds such as berberine, exhibiting various pharmacological activities including anti-inflammatory, anti-tumor, and lipid-regulating effects, and is widely utilized in the medical field (Li et al., 2022; Zhu et al., 2022). The growing global demand for natural pharmaceuticals has spurred extensive cultivation of this species across China. However, the expansion of *P. chinense* plantations has been accompanied by a persistent challenge: continuous monoculture, often implemented over multiple rotations, progressively degrades soil physicochemical properties, depletes nutrient pools, acidifies the rhizosphere, and induces physiological stress that manifests as diminished photosynthetic capacity and slowdown in growth (Jia et al., 2026). These interconnected belowground and aboveground constraints have become a primary bottleneck limiting the sustainable productivity of *P. chinense* cultivation systems (Xing et al., 2025). Agroforestry or intercropping (e.g., mixing with herbs, bamboo, or economic crops) is considered a robust approach to overcoming monoculture obstacles by optimizing the forest microenvironment, regulating soil nutrient cycling, and enhancing overall system stability through rational species combinations (Castle et al., 2022). For *P. chinense* empirical evidence regarding optimal intercropping partners remains scarce, and the mechanistic basis for differential intercropping outcomes has yet to be systematically dissected. A growing body of research has recognized that intercropping effects on plant performance are not solely mediated by aboveground interactions but are profoundly shaped by belowground processes occurring in the rhizosphere—the narrow zone of soil directly influenced by root activity (Veen et al., 2019; Yang X et al., 2021). The rhizosphere serves as a critical interface where root exudates, soil physicochemical properties, and microbial communities interact in a dynamic feedback loop (Mimmo et al., 2018). Intercropping alters this belowground environment by modifying root architecture, exudate composition, and interspecific root competition, which in turn induces shifts in soil pH, nutrient availability (e.g., nitrogen, phosphorus, and potassium), and organic matter content (Li et al., 2006; Hinsinger et al., 2011). Concurrently, these edaphic changes exert selective pressures on rhizosphere microbial assemblages, reshaping community composition, diversity, and functional potential (Berg and Smalla, 2009). Given that rhizosphere microbiomes are intimately linked to plant nutrient acquisition, phytohormone synthesis, and stress tolerance, it is plausible that intercropping-induced belowground modifications cascade upward to influence leaf-level physiological processes, particularly photosynthesis (Berendsen et al., 2012).

Current research on *P.chinense* has predominantly focused on single-properties analyses under monoculture plantation conditions, while integrated evidence that concurrently links rhizosphere soil dynamics, microbial community structure, and leaf photosynthetic physiological mechanisms within agroforestry systems remains critically scarce. Consequently, the extent to which intercropping-driven belowground alterations are functionally coupled with aboveground photosynthetic performance, and whether these couplings are mediated by specific microbial taxa or edaphic factors, remains largely unknown. Furthermore, existing investigations have predominantly targeted mature plantations of *P.chinense*, whereas the early-stage establishment phase—which is crucial for subsequent stand productivity and sustainable management—has received far less attention. This knowledge gap hampers the mechanistic understanding of intercropping benefits and impedes the evidence-based selection of optimal companion species for *P. chinense* cultivation. To address this gap, the present study selected five representative cultivation patterns commonly employed in agroforestry systems, all centered on *P. chinense*: intercropping with Magnolia officinalis (a broadleaf medicinal tree, T1), bamboo (Chimonobambusa spp., T2), Cryptomeria japonica (a coniferous timber tree, T3), Camellia sinensis (a perennial economic shrub, T4), and a monoculture control (T5). These companion species encompass contrasting life forms, functional traits, and ecological strategies, providing an ideal comparative platform to dissect species-specific intercropping effects. We systematically measured rhizosphere soil physicochemical properties, microbial community composition via, and leaf gas-exchange parameters. Additionally, we also measured the breast height diameter of *P.chinense*. we aimed to disentangle the coupling relationships among these three dimensions and reveal how belowground microenvironmental shifts covary with aboveground carbon assimilation. Collectively, our findings provide a basis for optimizing companion species selection in *P.chinense* intercropping systems and inform evidence-based agroforestry practices for sustainable medicinal tree cultivation.

## 2 Materials and methods

### 2.1 Study site description

The experimental site is located at a *P. chinense* planting base in Xintian Town, Yingjing County, Ya’an City, Sichuan province. The area experiences a subtropical monsoon climate, with a mean annual temperature of 16.8 °C, a mean annual precipitation of 1,918 mm, and an annual sunshine duration of 852.1 h. The soil type is mountain yellow earth, with a depth of 45–50 cm, the elevation is approximately 1,200 m, slope aspect (all plots facing semi-sunny slope), and slope position (mid-slope for all plots)”

### 2.2 Experimental design

This study relied on a previously established early-stage observation forest for *P. chinense*. Seedling afforestation was carried out in April 2023 after completing silvicultural tasks such as site preparation and weeding. The experiment was established using a randomized complete block design with three replicate blocks. *P. chinense* intercropping patterns were established: *P. chinense* + *M. officinalis* (T1), *P. chinense* + Chimonobambusa (T2), *P. chinense* + *C. japonica* (T3), *P. chinense* + *C. sinensis* (T4), and a *P. chinense* monoculture (T5) served as the control. Five cultivation patterns were assigned to plots within each block. Each plot measured 20 m × 20 m, and adjacent plots were separated by a 5 m buffer zone to minimize edge effects and cross-treatment interference. Within each plot, P. chinense saplings (2-year-old, uniform height of 60–80 cm) were planted at a fixed spacing of 2.0 m × 2.0 m (inter-row × intra-row) across all treatments. For intercropping treatments (T1–T4), companion plants were established in rows alternating with P. chinense rows. The distance between the P. chinense row and the adjacent companion plant row was uniformly set at 1.5 m to balance interspecific facilitation and competition. The intra-row spacing for companion species was adjusted according to their growth habits: 2.0 m for M. officinalis, 1.0 m for bamboo, 2.0 m for C. japonica, and 0.5 m for C. sinensis (inter-row spacing for all companion species was 1.5 m), and field management (irrigation, weeding, and pest control) throughout the experimental period. Collectively, these four species represent a gradient of functional contrasts—growth form (tree vs. shrub vs. bamboo vs. evergreen shrub), root architecture (deep vs. shallow), litter chemistry (broadleaf vs. coniferous vs. bamboo), and economic value—Field surveys and sampling for this study were conducted between May and August 2025, two years after afforestation. Three replicate standard plots (20 m × 20 m) were randomly established for each cultivation model in forest areas with highly consistent site conditions (slope, aspect, elevation, etc.) and prior management histories, totaling 15 standard plots for subsequent observation.

### 2.3 Soil sampling

Rhizosphere soil samples were collected exclusively from *P.chinense* individuals in each treatment plot (T1–T5) in August 2025. For each plot, five healthy and representative *P. chinense* trees of similar size were randomly selected. After removing the surface litter layer, soil was carefully excavated from the 0–20 cm depth within 5 cm of the main root surface. Soil adhering tightly to the root system (defined as rhizosphere soil) was collected using the root-shaking method. The rhizosphere soil collected from the five selected trees within the same plot was pooled and thoroughly homogenized to form one composite sample, thereby minimizing within-plot variability.

A total of 15 composite samples were obtained (3 replicate plots × 5 cultivation patterns). Each composite sample was passed through a 2 mm sieve to remove stones, coarse roots, and visible debris, and was then divided into two subsamples: one subsample was stored at 4 °C for subsequent analysis of soil physicochemical properties, while the other was stored at −80 °C for DNA extraction and high-throughput amplicon sequencing of microbial communities.

### 2.4 Soil physicochemical properties and high-throughput sequencing

Soil pH was measured using a pH meter in a 1:2.5 (v/v) soil-to-water suspension; soil organic carbon (SOC) content was determined using the potassium dichromate external heating oxidation method (Xu et al., 2019). Total nitrogen (N) was determined using the Kjeldahl method; total phosphorus (P) was determined by the molybdenum-antimony anti-colorimetric method after acid digestion. Total potassium (K) and medium/trace elements (Na, Mg, Ca, Fe, Mn, Cu, Zn) were quantitatively measured using an Inductively Coupled Plasma Optical Emission Spectrometer (ICP-OES) or an Atomic Absorption Spectrophotometer (AAS) following digestion with aqua regia (or nitric acid-perchloric acid) (Duan et al., 2023).

Total microbial DNA from the rhizosphere soil was extracted using a soil DNA extraction kit (Tiangen, China). After assessing DNA quality and precise quantification via 1% agarose gel electrophoresis, it was diluted to 1 ng·µL^−1^ as an amplification template. For bacterial communities, primers 341F (5′-CCTAYGGGRBGCASCAG-3′) and 806R (5′-GGACTACNNGGGTATCTAAT-3′) were used to amplify the V3-V4 hypervariable region of the 16S rRNA gene; for fungal communities, primers 1737F (5′-GGAAGTAAAAGTCGTAACAAGG-3′) and 2043R (5′-GCTGCGTTCTTCATCGATGC-3′) were used to amplify the ITS1-5F region. Following purification, PCR products were subjected to paired-end sequencing on the Illumina sequencing platform by Novogene Co., Ltd. (Beijing, China).

### 2.5 Measurement of tree diameter at breast height and photosynthetic parameters

Prior to the measurement of photosynthetic parameters, tree diameter at breast height (DBH) was recorded for all sampled *P.chinense* individuals within each treatment plot. DBH was defined as the stem diameter measured at 1.3 m above ground level, following standard forest inventory protocols (West, 2009). Photosynthetic parameters were measured exclusively on *P.chinense* individuals within each treatment plot (T1–T5). Measurements were conducted on fully expanded, healthy, and sun-exposed leaves from the middle canopy layer of *P. chinense*. For intercropping treatments (T1–T4), measurements were performed only on the target species (*P. chinense*), not on companion plants, to ensure consistent comparisons of intercropping effects on the focal species across all cultivation patterns. In gas exchange parameters of *P. chinense* leaves were measured using a portable photosynthesis system (LI-6800, LI-COR, USA) between 09:00 and 11:00 on clear days in mid-August 2025. Five plants with uniform growth were selected per pattern, and current-year functional leaves in the middle and upper parts of the crown, facing the sun and free of pests and diseases, were chosen for measurement. The leaf chamber conditions were set as follows: a red-blue light source with a constant photosynthetically active radiation (PAR) of 1,000 μmol m^−2^ s^−1^, a reference chamber CO_2_ concentration of 400 μmol mol^−1^, a leaf chamber temperature of 25 °C, and a relative humidity of 60%. Once photosynthetic readings stabilized, net photosynthetic rate (Pn), stomatal conductance (Gs), transpiration rate (Tr), and intercellular CO_2_ concentration (Ci) were recorded. Measurements were taken consecutively over 3 days to obtain average values (Yang et al., 2021).

### 2.6 Data processing and statistical analysis

Based on the raw sequences obtained from high-throughput sequencing, Following demultiplexing based on unique barcodes and trimming of barcodes, primers and adapters using Cutadapt (v 3.3) in Python (v 3.6.13), low-quality reads (Phred < 20) were filtered. Qualified paired-end reads were merged via DADA2, followed by *de novo* removal of chimeras. Sequences originating from negative controls were eliminated to remove background contamination. Taxonomic annotation was conducted against the SILVA database (v 138.1) and Unite database (v 9.0) with a confidence cutoff of 0.8. Operational Taxonomic Unit (OTU) clustering was performed using UPARSE software (at a 97% similarity threshold). The QIIME software invoked the BLAST algorithm to align representative sequences with the SILVA and UNITE databases to obtain taxonomic annotation information. Microbial α-diversity indices (including ACE, Chao1, Shannon, and Simpson indices) were calculated using QIIME software.

Statistical plotting and community analysis were performed using R software (v 3.3.1). 15 samples were applied to the following analysis. Venn diagrams were utilized to display shared and unique OTUs. Stacked relative abundance charts were plotted at the phylum level. Non-metric multidimensional scaling (NMDS) based on the Bray-Curtis distance matrix was performed to assess inter-group community differences. Furthermore, a permutational multivariate analysis of variance (PERMANOVA) was employed to evaluate the statistical significance of variations in soil bacterial and fungal community compositions. Redundancy analysis (RDA) was applied to explore the driving effects of soil physicochemical factors on the evolution of microbial community structure. SPSS 26.0 software was used for variance analysis, employing One-way ANOVA combined with Duncan’s multiple comparisons to test for significant differences among different cultivation patterns (*P* < 0.05). Pearson correlation analysis was utilized to examine the relationships among soil nutrients, photosynthetic parameters, and α-diversity indices, and heatmaps were generated using Origin 2022.

## 3 Results

### 3.1 Effects of different cultivation patterns on rhizosphere soil physicochemical properties

**Figure 1:**
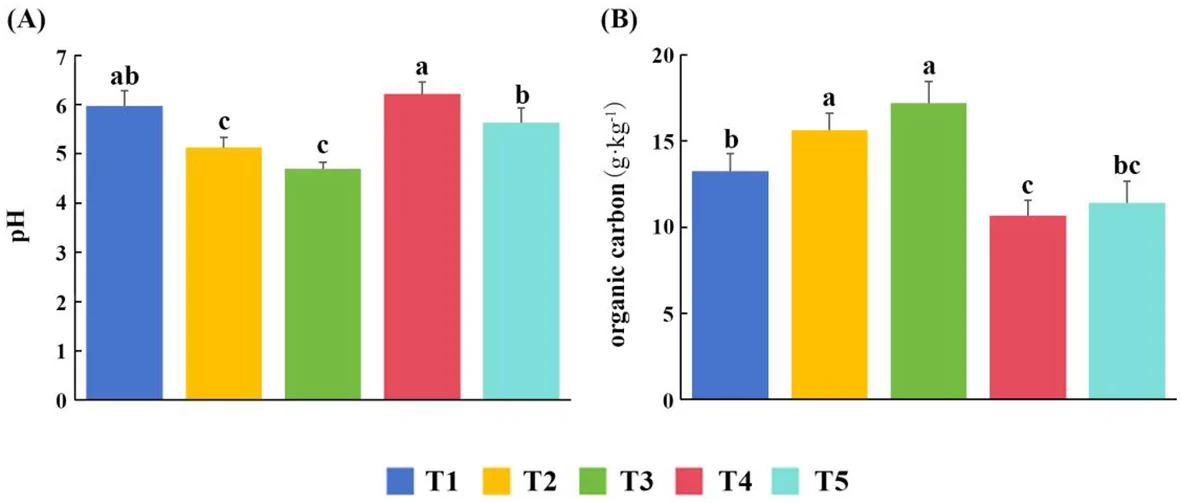
The pH and organic carbon fraction of rhizosphere soil in *P. Chinense* under different cultivation modes. Different lowercase letters indicate significant differences among different cultivation modes (*P* < 0.05).

**Figure 2:**
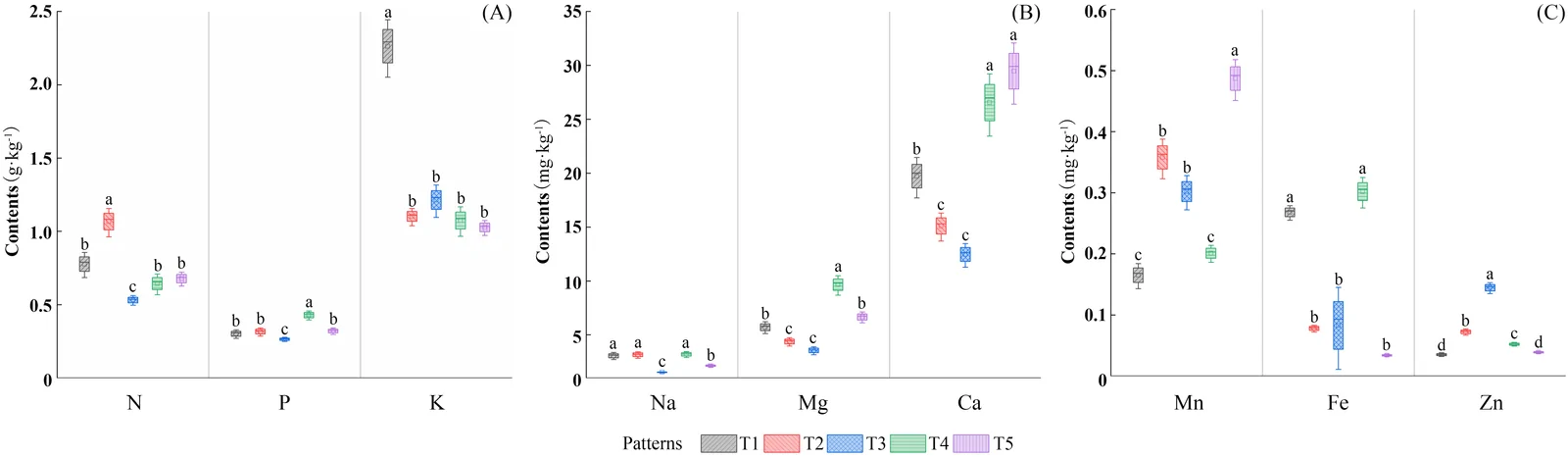
The mineral nutrients contents of rhizosphere soil in *P. Chinense* under different cultivation modes. Different lowercase letters indicate significant differences among different cultivation modes (*P* < 0.05).

Different cultivation patterns significantly affected the acid-base environment and nutrient pools of the *P. chinense* rhizosphere. Soil pH values ranged between 4.69 and 6.22 (**Figure 1A**). Notably, the *C. japonica* intercropping (T3) caused significant soil acidification (pH 4.69), accompanied by the highest level of organic carbon accumulation (17.21 g kg^−1^) (**Figure 1B**). In terms of macro- and micro-nutrients, the mixing of different species showed specific nutrient enrichment effects (**Figure 2**). Intercropping with bamboo (T2) and tea (T4) significantly elevated the levels of available nutrients in the soil. Total nitrogen (N) peaked in the T2 pattern (1.067 g kg^−1^), while total phosphorus (P) peaked in the T4 pattern (0.429 g kg^−1^) (*P* < 0.05). Furthermore, the rhizosphere microenvironment of the T4 pattern significantly enriched sodium (Na) and magnesium (Mg), whereas the pure forest (T5) exhibited an accumulation of calcium (Ca). The overall contents of trace elements (Fe, Mn, Cu, Zn) were low, but Cu and Zn showed significant increases in the T2 and T3 patterns (*P* < 0.01).

### 3.2 Diversity and structure of rhizosphere microbial communities

**Figure 3:**
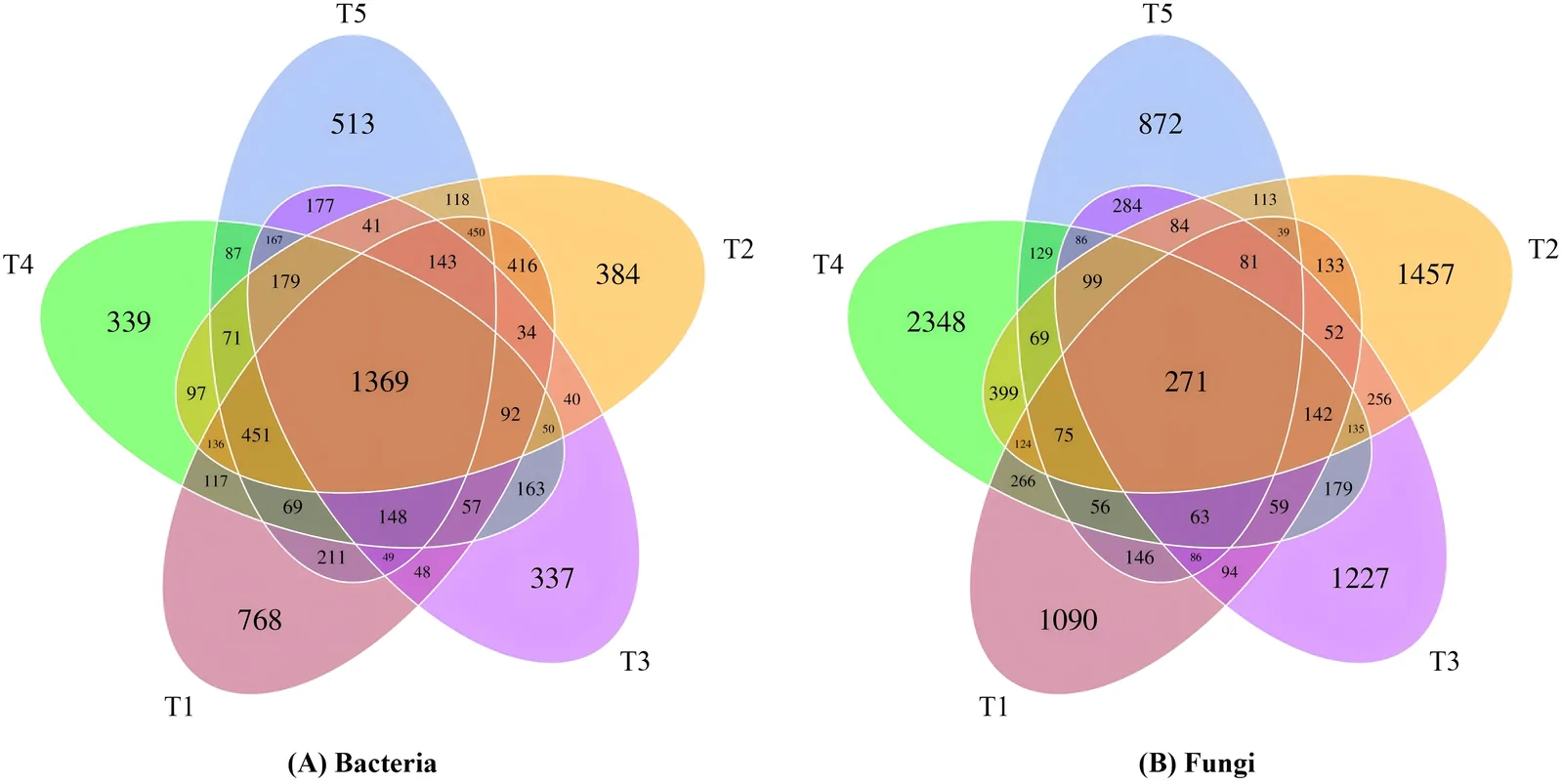
The Venn graphs of bacteria and fungi under different cultivation modes.

OTU clustering results showed (**Figure 3**) both commonalities and specificities in microbial species among the patterns; shared OTUs for bacterial and fungal communities accounted for 36.9% and 3.73% of the total, respectively, indicating a more pronounced response of the fungal community to changes in cultivation patterns. Regarding α-diversity, the T2 and T4 patterns exhibited higher niche capacities, with both bacterial and fungal Shannon and Chao1 indices at higher levels; conversely, microbial diversity was significantly restricted in the strongly acidic environment of the T3 pattern.

**Figure 4:**
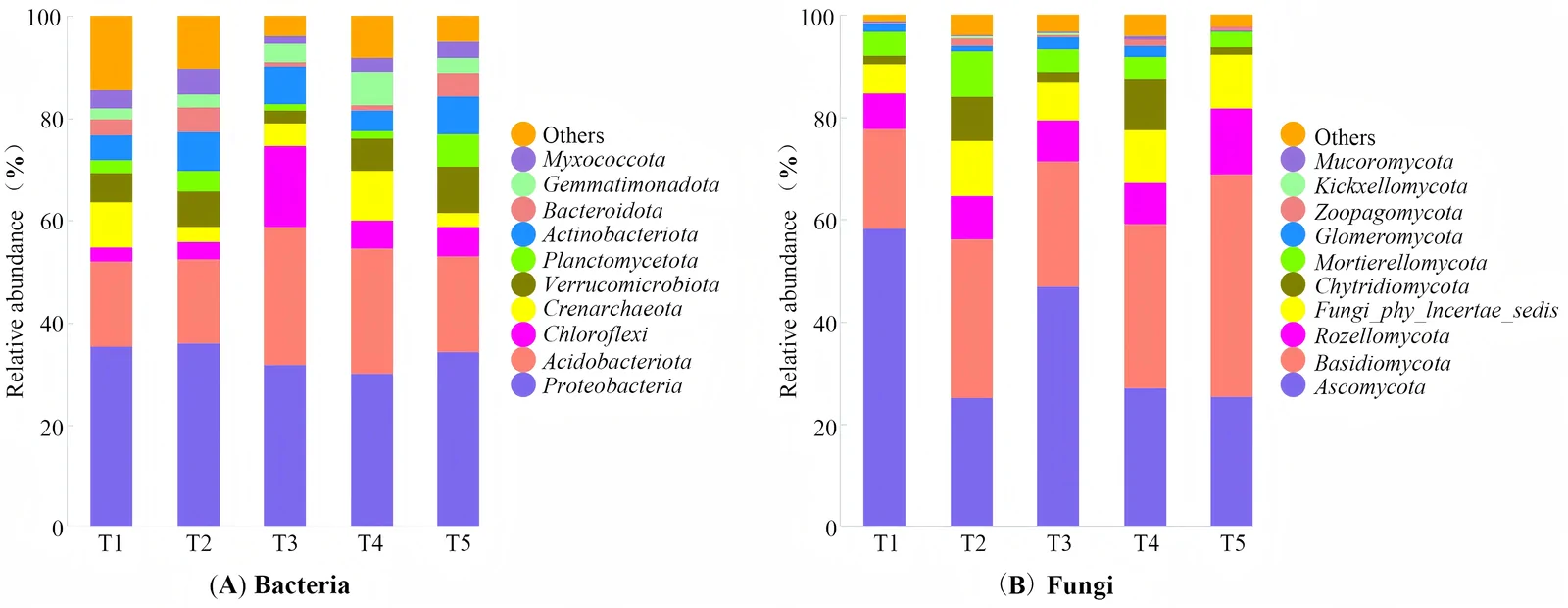
The relative abundance of soil bacteria and fungi at the phylum level under different cultivation modes.

**Figure 5:**
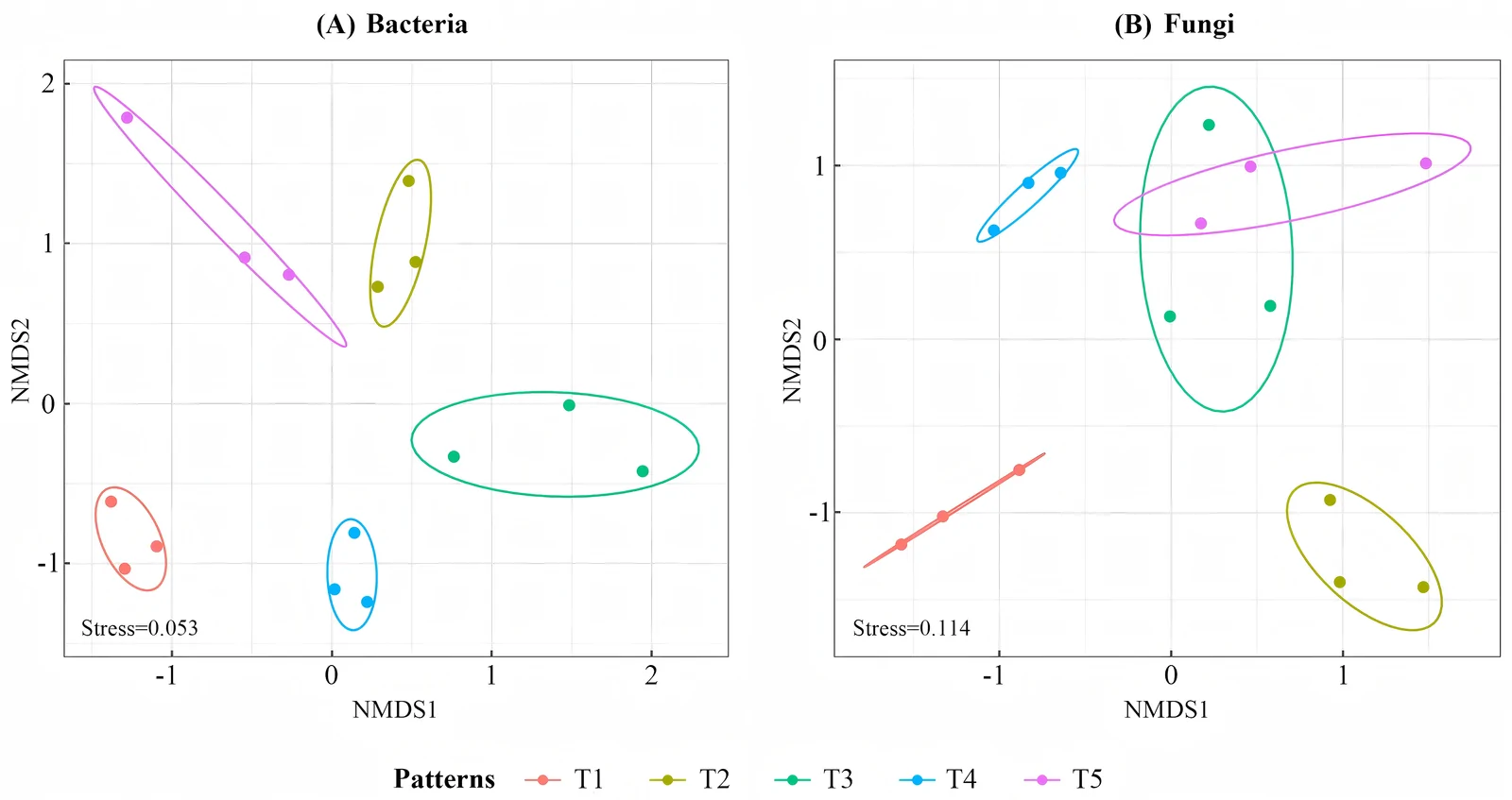
Non-metric multidimensional scaling (NMDS) analysis on soil microbial communities (Bacteria and Fungi) under different cultivation modes.

In terms of community composition (**Figure 4**), bacterial communities across all patterns were absolutely dominated by Proteobacteria and Acidobacteriota, but the relative abundance of Chloroflexi surged to 15.87% in the T3 pattern, significantly higher than in other patterns. Fungal communities were primarily dominated by Ascomycota and Basidiomycota, with *M. officinalis* (T1) and *C. japonica* (T3) intercropping significantly increasing the abundance of Ascomycota, while the monoculture (T5) highly enriched Basidiomycota. Non-metric multidimensional scaling (NMDS) further confirmed that, barring larger intra-group differences for fungal communities in T3 and T5, significant spatial separation occurred in both bacterial and fungal community structures under different cultivation patterns (**Figure 5**), indicating an obvious filtering effect of companion plants on rhizosphere microbial communities. PERMANOVA further confirmed significant divergence in microbial community compositions across the cultivation patterns. This structural variance was highly significant for both bacteria (R² = 0.478, P = 0.001) and fungi (R² = 0.612, P = 0.001).

### 3.3 Effects of different cultivation patterns on photosynthetic characteristics of *P. chinense*

**Figure 6:**
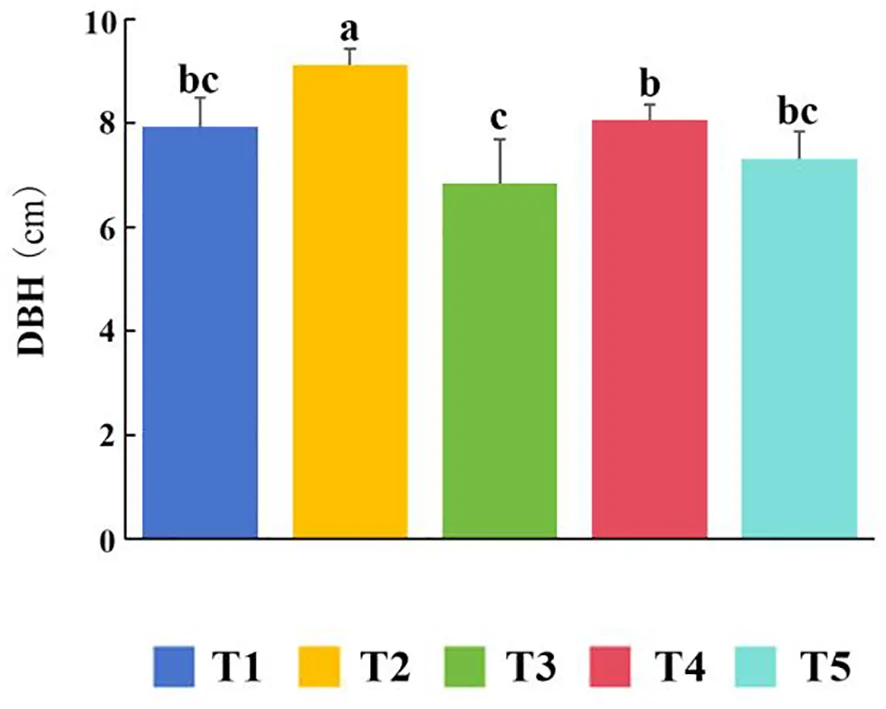
The DBH in *P. Chinense* under different cultivation modes.

**Figure 7:**
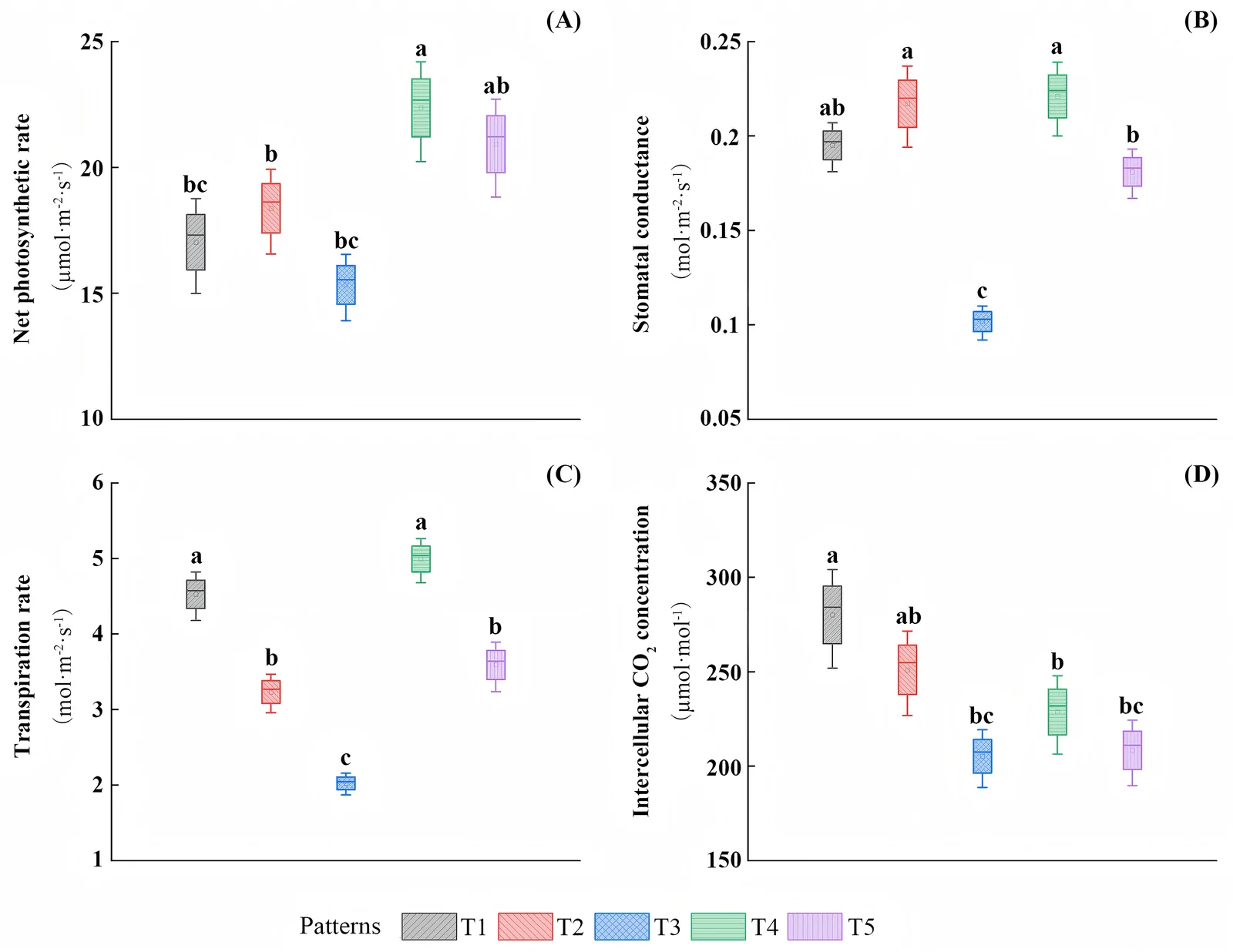
The Photosynthetic parameters of *P. chinense* under different cultivation patterns. Different lowercase letters indicate significant differences among different cultivation modes (*P* < 0.05).

Intercropping exerted significant effects on the photosynthetic physiological and Diameter at Breast Height (DBH) performance of *P.chinense* (*P* < 0.05) (**Figure 6**). T2 exhibited the optimal diameter at DBH, which was significantly greater than that of all other cultivation patterns (**Figure 6**) (*P* < 0.05). The T4 and T2 patterns have high net photosynthetic rates (Pn) and stochastic conductivity (Gs) Photosynthetic performance peaked under the tea intercropping (T4) pattern, with net photosynthetic rate (Pn), stomatal conductance (Gs), and transpiration rate (Tr) being significantly higher than in other patterns (*P* < 0.05). The Pn of the monoculture (T5) and bamboo intercropping (T2) ranked next. In contrast, *C. japonica* intercropping (T3) severely inhibited the photosynthetic activity of *P. chinense*, reducing all gas exchange parameters to their lowest levels. Regarding intercellular CO_2_ concentration (Ci), the T1 pattern showed significantly higher Ci than the other groups, suggesting its lower Pn might primarily be constrained by non-stomatal factors (**Figure 7**).

### 3.4 Correlation and driving factor analysis of soil physicochemical factors, microbial communities, and photosynthetic parameters

**Figure 8:**
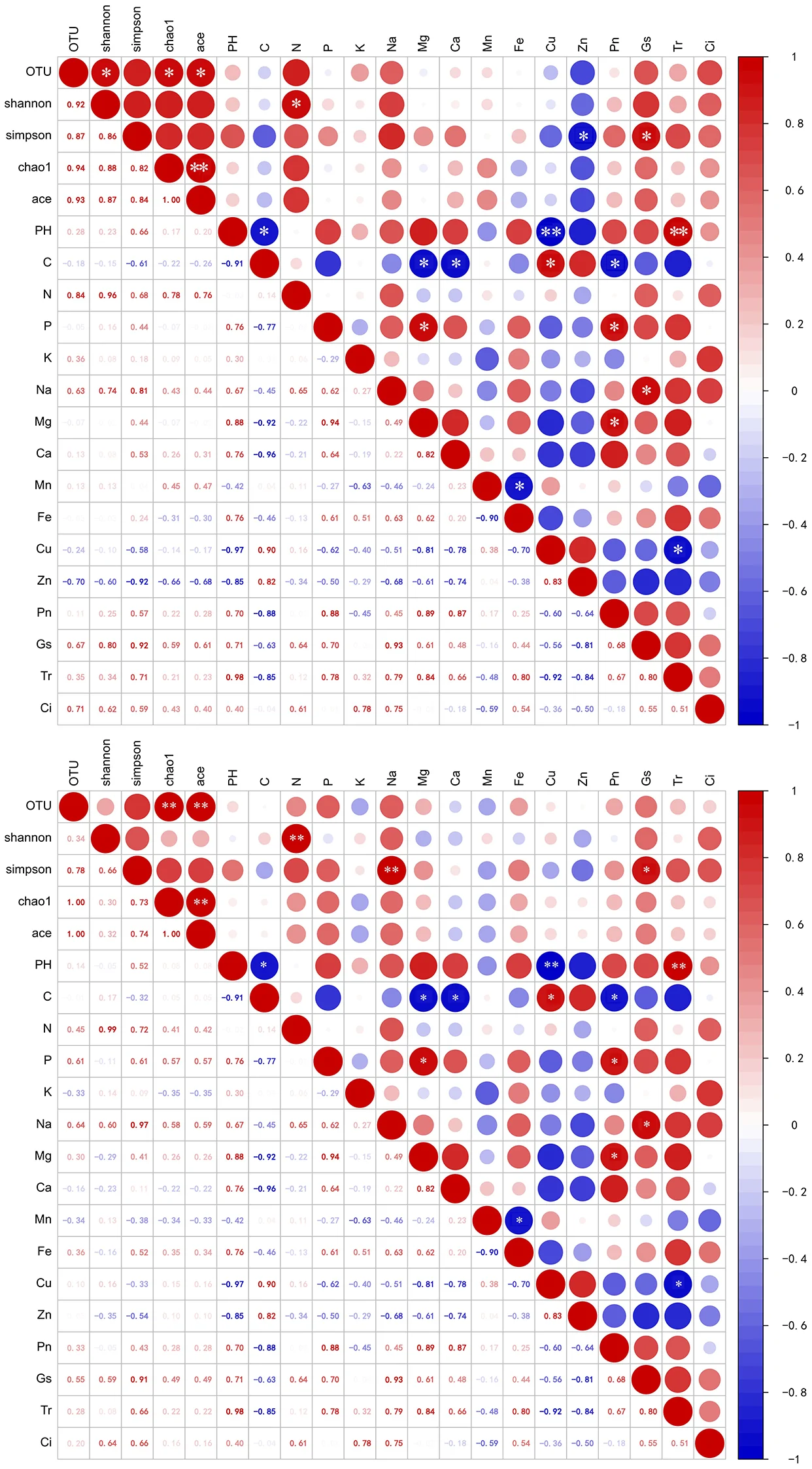
Heatmap of correlations between soil nutrients, photosynthetic parameters, and rhizosphere microbial diversity indices (Above: Bacteria; Below: Fungi). ** indicate significant correlation at 0.01 level; * indicate significant correlation at 0.05 level.

To elucidate the intrinsic linkages within the microecosystem, pearson correlation analysis was conducted on soil factors, photosynthetic parameters, and microbial diversity (**Figure 8**). The results demonstrated that plant photosynthetic capacity was highly coupled with specific soil nutrients: Pn was significantly and positively correlated with soil P and Mg contents (*P* < 0.05), but negatively correlated with soil C content; Gs was significantly and positively correlated with soil Na content. At the microbial level, an increase in soil N content significantly promoted the Shannon diversity of bacteria and fungi (*P* < 0.05), while Gs, representing photosynthetic activity, also exhibited a positive association with the Simpson dominance index of bacteria and fungi.

**Figure 9:**
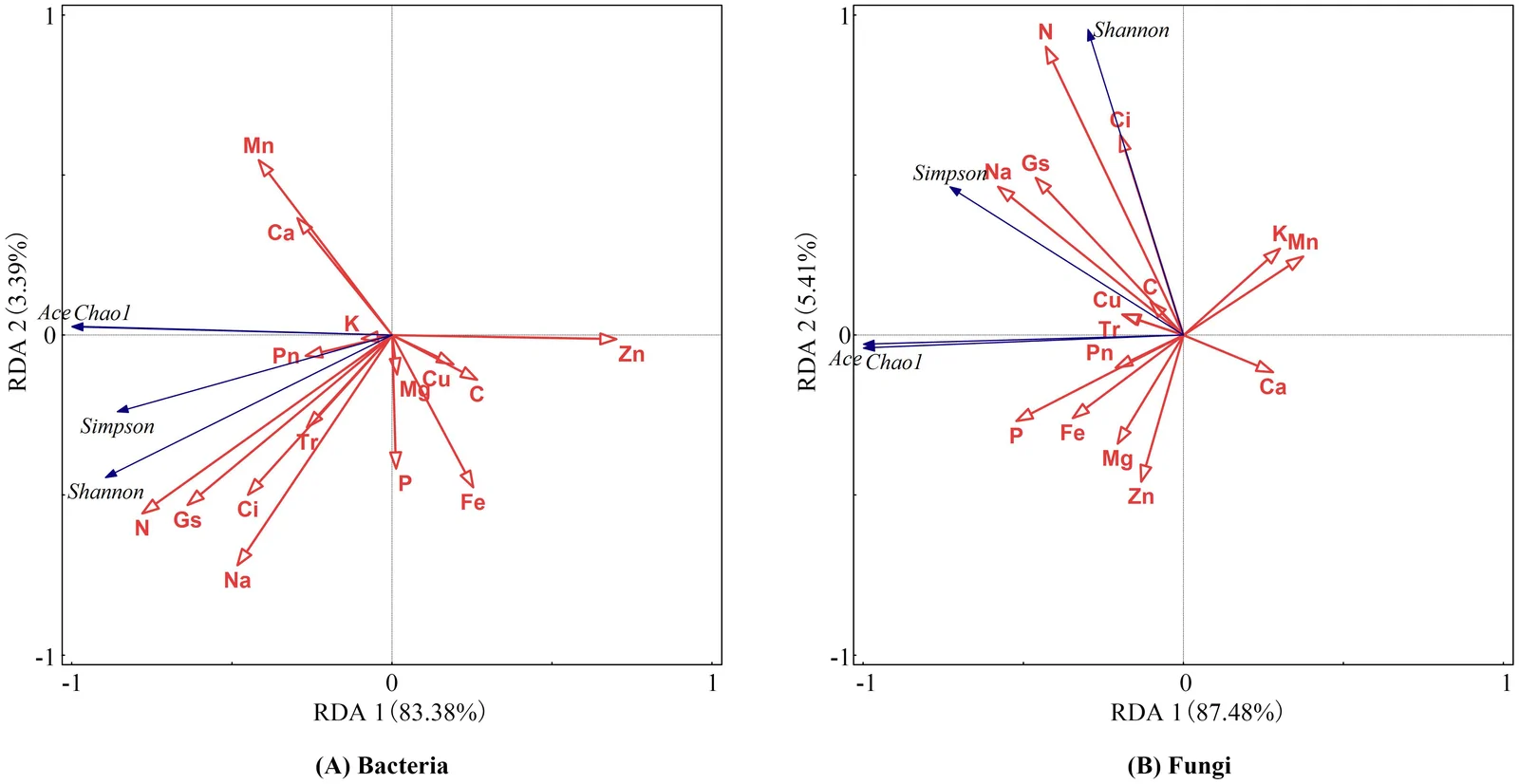
Redundancy analysis (RDA) illustrating the relationships between the rhizosphere microbial community structure (at the OTU level) and both soil physicochemical and photosynthetic parameters in *P. chinense*.

RDA further quantified the degree of impact of physicochemical factors on community structure (**Figure 9**). The first two RDA axes explained the vast majority of total community variation (85.77% for bacteria, 92.89% for fungi). Notably, soil N and Na were identified as the key environmental factors affecting changes in the structure of rhizosphere fungal and bacterial communities of *P. chinense*.

## 4 Discussion

This study demonstrates that the introduction of different companion plants significantly altered the physicochemical microenvironment of the *P. chinense* rhizosphere. Soil pH and nutrient pools exhibited strong, species-specific differentiation across the intercropping models. Intercropping with *C. japonica* (T3) resulted in severe soil acidification (pH 4.69), This phenomenon aligns with classical biogeochemical theories regarding coniferous litter traits (Augusto et al., 2015). Coniferous needles possess high lignin-to-nitrogen ratios. They are also exceptionally rich in recalcitrant secondary metabolites, including resins, tannins, and complex polyphenols (Kraus et al., 2003). During decomposition, the leaching of these acidic compounds releases abundant low-molecular-weight organic acids (Tanikawa et al., 2018). Distinct intercropping combinations exhibited specific nutrient enrichment effects. Intercropping with bamboo and tea demonstrated profound nutrient activation, effectively reversing this soil degradation trajectory. Total nitrogen (N) reached its maximum in the bamboo-intercropped treatment, whereas total phosphorus (P) peaked in the tea-intercropped treatment. This species-specific nutrient enrichment can be attributed to differences in root architecture, exudate composition, and nutrient acquisition strategies among the companion species. Bamboo characterized by an extensive root system and a potential capacity to recruit nitrogen-fixing microbial communities, may enhance rhizosphere N levels through increased N fixation or reduced N leaching (Zhang et al., 2025; Kaushal et al., 2020). Furthermore, the high-frequency turnover of bamboo fine roots, combined with a relatively low C/N ratio in its litter return, accelerates soil organic nitrogen mineralization. This process prevents nitrogen immobilization (Xu et al., 2022; Tripathi and Singh, 1992). Tea (C.sinensis), as an acidophilic plant, may mobilize previously fixed P through root-induced acidification or stimulate organic P mineralization, thereby elevating total P pools (Peng et al., 2023; Morita et al., 2011).

At the phylum level, bacterial communities across all treatments were overwhelmingly dominated by Proteobacteria and Acidobacteriota, which is consistent with their well-documented prevalence in forest soils worldwide (Fierer et al., 2007). However, the relative abundance of Chloroflexi surged to 15.87% in the T3 pattern, substantially exceeding that in other treatments. This enrichment is noteworthy, as Chloroflexi have been frequently associated with nutrient-poor or stressed environments, and certain members of this phylum are capable of participating in carbon fixation under oligotrophic conditions (Xia et al., 2020). For fungal communities, Ascomycota and Basidiomycota were the dominant phyla across all patterns, consistent with their ubiquitous roles as decomposers of complex plant litter in terrestrial ecosystems (Tedersoo et al., 2014). Intercropping with M. officinalis (T1) and C. japonica (T3) significantly increased the relative abundance of Ascomycota, a phylum encompassing many saprotrophic taxa capable of degrading recalcitrant substrates under acidic conditions. Our research reveals that the T2 and T4 intercropping patterns significantly enhanced the α-diversity (both Shannon and Chao1 indices) of rhizosphere bacterial and fungal communities, Indicating that intercropping reshapes the rhizosphere microbiome (Berg and Smalla, 2009). Furthermore, the taxonomic shifts observed in our study provide biological evidence for the enhanced nutrient activation. For instance, the synergistic intercropping patterns (T2 and T4) significantly enriched specific keystone bacterial genera. These taxa are widely recognized in the literature for their robust capacity to secrete low-molecular-weight organic acids and extracellular phosphatases, which effectively solubilize fixed inorganic phosphorus and accelerate organic matter mineralization (Li et al., 2023). In contrast, the T3 pattern (C. japonica intercropping) exhibited a marked suppression of microbial diversity, which coincided with the most severe soil acidification (pH 4.69) and the highest SOC accumulation observed in this study. The strongly acidic conditions likely imposed environmental filtering that excluded acid-sensitive taxa, while favoring only those microorganisms adapted to low-pH habitats (Rousk et al., 2010). This diversity decline under T3 suggests that the pronounced acidification outweighed any potential positive effects of organic carbon enrichment on microbial community richness. Proteobacteria are deeply involved in the degradation of complex organic matter and play pivotal roles in global carbon and nitrogen cycling (Spain et al., 2009). Conversely, the T3 pattern developed an extreme microenvironment. This system suffered from strong acidity, severe base cation depletion, and overall nutrient deprivation. Consequently, this microenvironment induced potent environmental stress (Rousk et al., 2010). This extreme selective pressure forced the community to undergo a successional shift toward highly tolerant, slow-growing oligotrophic (K-strategist-like) taxa (Fierer et al., 2007), such as Chloroflexi, which are equipped with extensive stress-response gene repertoires allowing them to scavenge sparse nutrients in hostile environments (Hug et al., 2013). Furthermore, fungal communities shifted dramatically across the different cultivation models. This structural turnover provides profound insights into organic matter decomposition dynamics (Baldrian, 2017).

Intercropping with *M. officinalis* and *C. japonica* (T1, T3) significantly enriched Ascomycota, which generally act as primary, early-stage decomposers adept at rapidly utilizing easily degradable, labile carbon sources (such as simple sugars and hemicellulose) (Voříšková and Baldrian, 2013). However, the *P. chinense* monoculture (T5) was highly and anomalously dominated by Basidiomycota. Basidiomycota are recognized as late-stage decomposers, evolutionarily equipped with potent, specialized oxidative enzymatic machinery (including lignin peroxidases, manganese peroxidases, and laccases) required to break down complex aromatic polymers (Lundell et al., 2010; Sinsabaugh, 2010). The pure forest exhibits an extreme enrichment of Basidiomycota. This phenomenon suggests a profound microecological crisis. Specifically, recalcitrant lignocellulosic debris accumulates massively in the soil. Microbial degradation of this debris is likely restricted in the short term. Non-metric multidimensional scaling (NMDS) analysis revealed significant spatial separation of both bacterial and fungal community structures under different cultivation patterns, with the exception of relatively large intra-group variations observed for fungal communities in T3 and T5. This overarching pattern indicates a pronounced filtering effect of companion plants on rhizosphere microbial communities, whereby each intercropping system selected for a distinct microbial assemblage adapted to its specific rhizosphere conditions. Collectively, our findings demonstrate that the selection of intercropping partners exerts a deterministic influence on the assembly and composition of rhizosphere microbial communities in *P. chinense* plantations. The T2 and T4 patterns emerged as favorable systems for maintaining high microbial diversity and niche differentiation, likely due to the complementary root architectures and exudate chemistries of bamboo and tea, respectively. In contrast, the T3 pattern, while promoting SOC sequestration, imposed strong environmental filtering that reduced microbial diversity and selected for a specialized, acid-adapted community. This study reveals a powerful ecological feedback mechanism. Belowground soil nutrients and microbial diversity optimize synergistically. This specific rhizosphere environment profoundly improves the aboveground photosynthetic carbon assimilation capacity of *P. chinense*. Correlation analysis confirmed that the net photosynthetic rate (Pn) was significantly and positively correlated with soil P and Mg availability. From a stringent biochemical and photophysiological perspective, P is not merely the structural backbone of nucleic acids and thylakoid biomembranes (Veneklaas et al., 2012); At the same time, magnesium constitutes the central excitation atom in the chlorophyll porphyrin ring. The availability of P and Mg improves synergistically. This synergy solidifies the physical structural integrity of chloroplasts. Consequently, it maximizes the photochemical transduction efficiency of the photosynthetic apparatus (Carstensen et al., 2018; Tränkner et al., 2018). This fundamental biochemical alleviation explains unequivocally why the T4 pattern possessed the optimal, highest-performing photosynthetic efficiency. Even more novel and compelling is our finding that stomatal conductance (Gs) was positively correlated not only with soil total Na content but also closely coupled with the microbial Simpson dominance index. This implies a crucial and highly adaptive cross-scale osmotic regulation strategy in complex, competitive forest environments. It functions as a highly efficient osmoticum (Kronzucker et al., 2013). Guard cells rapidly compartmentalize this ion within their vacuoles. This ensures appropriate stomatal opening and a sufficient, uninterrupted intercellular CO_2_ supply, thereby effectively overcoming the severe stomatal limitations that predominantly suppress carbon fixation in pure forests. Stomatal conductance and net photosynthesis increased concurrently. This dual enhancement drives a robust transpiration pull. Consequently, it indicates a highly active carbon metabolism across the whole plant (Kuzyakov and Gavrichkova, 2010).

These complex cross-kingdom interactions demonstrate that the *P. chinense* intercropping system constructs a highly efficient and self-sustaining positive feedback loop within a holobiont framework. The system maintains this loop through a coordinated sequence involving nutrient activation, stomatal optimization, photosynthetic enhancement, and subsequent carbon source replenishment (Philippot et al., 2015).

Our findings should be interpreted as treatment-induced differentiations at a specific developmental stage rather than persistent or seasonally invariant patterns. Future multi-temporal investigations (e.g., seasonal sampling across multiple years) are required to assess the temporal stability of these belowground differentiations and their cascading effects on aboveground physiology, particularly given that *P. chinense* is a long-rotation species where intercropping benefits may emerge or shift over successional time. This study provides critical empirical insights into the sustainable management of *P. chinense*. Ultimately, our findings demonstrate that introducing specific companion plants, particularly bamboo and tea, offers a practical ecological strategy to overcome continuous monoculture obstacles. By reshaping the rhizosphere microbiome and enriching targeted soil nutrients (such as N, P, Mg, and Na), these synergistic intercropping regimes create an optimized belowground microenvironment. This microecological improvement is strongly associated with the alleviation of aboveground stomatal limitations and the enhancement of whole-plant carbon assimilation.

## Data Availability

The original contributions presented in the study are publicly available. This data can be found here: https://www.ncbi.nlm.nih.gov/ bioproject under the accession number PRJNA1517765.
